# Gray Matter Changes in Subjects at High Risk for Developing Psychosis and First-Episode Schizophrenia: A Voxel-Based Structural MRI Study

**DOI:** 10.3389/fpsyt.2013.00016

**Published:** 2013-03-18

**Authors:** Kazue Nakamura, Tsutomu Takahashi, Kiyotaka Nemoto, Atsushi Furuichi, Shimako Nishiyama, Yumiko Nakamura, Eiji Ikeda, Mikio Kido, Kyo Noguchi, Hikaru Seto, Michio Suzuki

**Affiliations:** ^1^Department of Neuropsychiatry, Graduate School of Medicine and Pharmaceutical Sciences, University of ToyamaToyama, Japan; ^2^Core Research for Evolutional Science and Technology, Japan Science and Technology CorporationTokyo, Japan; ^3^Department of Psychiatry, Graduate School of Comprehensive Human Sciences, University of TsukubaTsukuba, Ibaraki, Japan; ^4^Department of Radiology, Graduate School of Medicine and Pharmaceutical Sciences, University of ToyamaToyama, Japan

**Keywords:** schizophrenia, psychosis, high risk, MRI, cingulate gyrus

## Abstract

**Objectives:** The aim of the present study was to use a voxel-based magnetic resonance imaging method to investigate the neuroanatomical characteristics in subjects at high risk of developing psychosis compared with those of healthy controls and first-episode schizophrenia patients.

**Methods:** This study included 14 subjects with at-risk mental state (ARMS), 34 patients with first-episode schizophrenia, and 51 healthy controls. We used voxel-based morphometry with the Diffeomorphic Anatomical Registration through Exponentiated Lie Algebra tools to investigate the whole-brain difference in gray matter volume among the three groups.

**Results:** Compared with the healthy controls, the schizophrenia patients showed significant gray matter reduction in the left anterior cingulate gyrus. There was no significant difference in the gray matter volume between the ARMS and other groups.

**Conclusion:** The present study suggests that alteration of the anterior cingulate gyrus may be associated with development of frank psychosis. Further studies with a larger ARMS subjects would be required to examine the potential role of neuroimaging methods in the prediction of future transition into psychosis.

## Introduction

Neuroimaging studies have demonstrated subtle but widespread brain structural alterations, such as volume reduction of fronto-temporo-limbic regions as well as enlarged lateral and third ventricles, in first-episode schizophrenia (Steen et al., 2006; Vita et al., 2006; Ellison-Wright et al., 2008), which are not due to illness chronicity and antipsychotic medication. Recent prospective longitudinal magnetic resonance imaging (MRI) studies, including our own data showing progressive gray matter reduction of the temporal region (approximately 2–3% per year) (Takahashi et al., 2010, 2011), further revealed progressive brain structural change and its relationship to clinical course or outcome in first-episode schizophrenia (Andreasen et al., 2011). These longitudinal findings might be consistent with the clinical observation that a long duration of untreated psychosis (DUP), which could lead to severe brain pathological changes during the early illness stage (Lappin et al., 2006; Takahashi et al., 2007), is related to poor outcome of schizophrenia patients (Marshall et al., 2005; Perkins et al., 2005). Examining potential neurobiological markers that predate the onset of psychosis might lead to appropriate early intervention and thus prevent deterioration of social function and the progression of structural brain alterations.

It is not yet clear at which illness stage brain abnormalities occur in schizophrenia. Subjects with at-risk mental state (ARMS), who exhibit prodromal-like symptoms and have an increased risk of developing psychosis (Yung et al., 2003), might share disease vulnerability as well as brain morphological changes with patients with overt schizophrenia. Subjects with ARMS are heterogeneous on the basis of their outcome, as only about 36% of them develop psychosis during 3-year follow-up (Fusar-Poli et al., 2012). Previous MRI studies using voxel-based morphometry (VBM), which allows automated whole-brain analysis, revealed more severe gray matter reduction predominantly in the fronto-temporo-limbic regions in ARMS subjects with later transition than in those without (Pantelis et al., 2003; Borgwardt et al., 2007; Fusar-Poli et al., 2011). More specifically, Fornito et al. (2008) revealed that baseline differences in the anterior cingulate cortical thickness distinguished between ARMS with and without later transition, but they did not directly compare ARMS subjects and patients with overt psychosis.

This voxel-based MRI study aimed to investigate the nature of neuroanatomical abnormalities in high-risk subjects compared with both healthy controls and first-episode schizophrenia patients. On the basis of previous neuroimaging findings, we predicted that both first-episode schizophrenia and ARMS subjects, especially those with later transition, would show brain morphological changes in fronto-temporo-limbic regions compared with healthy subjects.

## Materials and Methods

### Participants

Fourteen individuals (10 males and 4 females) defined as ARMS for psychosis were recruited from the Consultation Support Service in Toyama (CAST), which was launched in 2006 as a specialized clinical setting to study and treat young persons (aged 15–30 years) at risk of developing psychosis (Mizuno et al., 2009). The subjects with ARMS were diagnosed according to the Comprehensive Assessment of ARMS (CAARMS) (Yung et al., 2004); they were characterized by one or more of the following: (1) attenuated psychotic symptoms; (2) brief, limited intermittent psychotic symptoms with spontaneous resolution; or (3) family history of psychosis in first-degree relatives or a personal history of schizotypal personality disorder accompanied by a decline in general functioning. Their clinical symptoms were assessed using the Scale for the Assessment of Negative Symptoms (SANS) (Andreasen, 1983) and the Scale for the Assessment of Positive Symptoms (SAPS) (Andreasen, 1984) at the time of scanning. Eleven ARMS subjects were neuroleptic-naïve at scanning, but two subjects were treated with atypical neuroleptics and one was receiving sulpiride. Their duration of medication use was shorter than 2 weeks for atypical neuroleptics and shorter than 6 months for sulpiride. They were also receiving benzodiazepines (*N* = 2), antidepressants (*N* = 1), and tandospirone (*N* = 3).

Thirty-four patients with first-episode schizophrenia (20 males and 14 females), who met the ICD-10 research criteria (World Health Organization, 1993), were recruited from the inpatient and outpatient clinics of the Department of Neuropsychiatry, Toyama University Hospital. The patients were diagnosed following structured clinical interviews by experienced psychiatrists using the Comprehensive Assessment of Symptoms and History (CASH; Andreasen et al., 1992). Their durations from manifestations of overt psychotic symptoms were shorter than 1 year. Their clinical symptoms were assessed using SANS and SAPS at the time of scanning. Thirty-three patients were receiving neuroleptic medication at the time of scanning; 2 patients were treated with typical neuroleptics, 26 were receiving atypical neuroleptics, 5 were taking both typical and atypical neuroleptics, and 1 patient was neuroleptic-free. They were also receiving anticholinergic drugs (*N* = 8), benzodiazepines (*N* = 9), antidepressants (*N* = 1), carbamazepine (*N* = 1), and lithium carbonate (*N* = 3).

Exclusion criteria for ARMS subjects and schizophrenia patients were other neurological diseases, past or present regular alcohol abuse, and/or consumption of illicit drugs as reported by the study participants and/or the patients’ records, as well as past head trauma with loss of consciousness or electro-convulsive treatment.

The control subjects consisted of 51 healthy volunteers (30 males and 21 females) recruited from members of the community, hospital staff, and university students. They were given a questionnaire consisting of 15 items concerning their personal (13 items; including a history of obstetric complications, substantial head injury, seizures, neurological or psychiatric diseases, impaired thyroid function, hypertension, diabetes, and substance use) and family (2 items) histories of illness. They did not have any personal or family history of psychiatric illness in their first-degree relatives. This study was approved by the ethics committee of Toyama University. Written informed consent was obtained from all subjects prior to study participation.

### MRI acquisition

Magnetic resonance images were obtained by utilizing a 1.5-T Magnetom Vision (Siemens Medical System, Inc., Erlangen, Germany) with a three-dimensional gradient-echo sequence FLASH (fast low-angle shots) yielding 160–180 contiguous T1-weighted slices of 1.0-mm thickness in the sagittal plane. The imaging parameters were as follows: TR = 24 ms; TE = 5 ms; flip angle = 40°; field of view = 256 mm; and matrix size = 256 × 256 pixels. The voxel size was 1.0 mm × 1.0 mm × 1.0 mm. All scans in the patient and control groups were acquired in the same system with the same protocol.

### MRI data processing

All T1-weighted MRI data were first converted from the Dicom format to the NIFTI format and then processed using Statistical Parametric Mapping 8 (SPM8, Wellcome Institute of Neurology, University College London, UK, http://www.fil.ion.ucl.ac.uk/spm) running under MATLAB R2008b (The MathWorks Inc., USA).

The unified segmentation model consisting of spatial normalization, bias field correction, and tissue segmentation was performed in order to improve the quality of data preprocessing (Ashburner and Friston, 2005). Tissue probability maps were registered to the subject’s data, and final tissue probability maps were derived from prior maps with the use of a combination with tissue probabilities based on the voxel intensity. To make the processed data more accurate, we used the Diffeomorphic Anatomical Registration through Exponentiated Lie Algebra (DARTEL) (Ashburner, 2007; Ashburner and Friston, 2009; Klein et al., 2009) tool in SPM8. DARTEL is not integrated into the segmentation model and requires the input of gray matter tissue maps produced by unified segmentation. This algorithm records inter-subject images using diffeomorphisms, which preserve the object properties through deformations, twistings, and stretchings, and archives a more accurate inter-subject registration. Because DARTEL produces a more accurate registration, it improves the sensitivity of finding and localizing differences between groups in terms of the gray matter volume. Registered tissue maps were transformed to the stereotactic space of the Montreal Neurological Institute (MNI) and multiplied with the Jacobian determinants of the deformations in order to preserve the volume of tissue in each structure. Finally, the modulated, warped tissue maps were then written with an isotropic voxel resolution of 1.5 mm^3^ and smoothed with a 10-mm Full-Width Half-Maximum (FWHM) Gaussian kernel (Salmond et al., 2002; Jones et al., 2005).

### Statistical analysis

#### Demographic data

Group differences in age, educational level, parental educational level, and intracranial volume (ICV) were examined with one-way analysis of variance (ANOVA) and *post hoc* Scheffé’s test. Group differences in terms of gender were tested with Chi-square tests. The level of statistical significance was defined as *p* < 0.05 (two-tailed). Statistical analyses were performed with Statistica, version 06J for Windows (StatSoft Japan Inc., Tokyo, Japan).

#### Voxel-based analysis of gray matter volume

Gray matter volume differences between the ARMS subjects, schizophrenia patients, and healthy controls were analyzed using two-sample *t*-tests implemented in the general linear model approach of SPM8 with age and ICV as nuisance covariates. We used cluster level inference (the extent of contiguous clusters of individual significant voxels) for determination of statistical significance (Meisenzahl et al., 2008). Because cluster size distribution varies according to local smoothness, the cluster sizes in this study were adjusted according to the local smoothness within the framework of the Random Field Theory (RFT) (Worsley et al., 1999; Hayasaka et al., 2004). Our statistical inference was performed at the cluster level by assessing the SPM{t} images by the non-stationary cluster extent correction (Hayasaka et al., 2004), which has been reported to be robust when MRI experiments fulfill (1) degrees of freedom > 30 and (2) image smoothness (FWHM) > 3 × voxel sampling resolution (Hayasaka et al., 2004), as in this study. The cluster-defining threshold was set to *p* < 0.001. Then, a family-wise error-corrected (FWE) cluster size threshold of *p* < 0.05 was applied to account for multiple comparisons of the results (corrected cluster sizes). Finally, cluster sizes were adjusted for smoothness non-uniformity using the VBM8 toolbox (Gaser, 2009), which implements the methodology of Hayasaka et al. (2004).

Voxel coordinates are given as an indication of location in a standardized brain. Voxels were localized in MNI space and transformed into Talairach and Tournoux coordinates (Talairach and Tournoux, 1988).

## Results

### Demographic data

Table 1 shows demographic and clinical data of the subjects in this study. Groups were matched for gender, parental education, and ICV. However, the controls (*p* < 0.001) and schizophrenia patients (*p* < 0.001) were older than the ARMS subjects. The controls had a higher educational level than the other two groups (*p* < 0.001) and the schizophrenia patients had a higher educational level than the ARMS subjects (*p* = 0.004).

**Table 1:** **Clinical and demographic characteristics^a^**.

Characteristic	ARMS (*N* = 14)	Schizophrenia (*N* = 34)	Healthy control (*N* = 51)
Gender (male/female)	10/4	20/14	30/21
Age (years)^b^	18.9 (1.4)	24.7 (5.5)	23.9 (1.8)
Educational level (years)^c^	11.6 (1.4)	13.5 (2.0)	16.0 (1.7)
Parental educational level (years)	13.7 (1.4)	13.3 (1.7)	14.1 (2.2)
Age at onset (years)	N/A	23.3 (5.4)	N/A
Duration of medication (months)	0.43 (1.6)	1.7 (1.8)	N/A
Drug (mg/day, haloperidol equivalent)^d^	0.55 (1.1)	6.3 (6.5)	N/A
Intracranial volume (cm^3^)	1557.8 (130.0)	1602.1 (150.7)	1573.6 (143.0)

*^a^Values given as mean (SD)*.

*^b^Significant difference between groups*.

*^c^Significant difference between groups*.

*^d^The different typical and atypical neuroleptic dosages were converted into haloperidol equivalents using the guidelines of Toru (2001)*.

### Voxel-based analysis of gray matter volume

**Figure 1:**
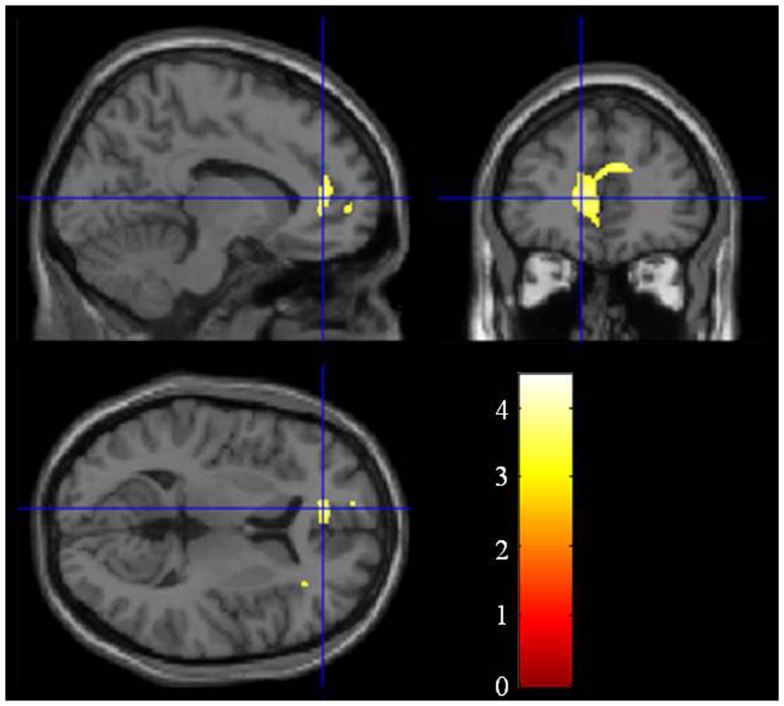
**Group difference of the gray matter between the schizophrenia patients and the healthy controls**. The cluster in which the schizophrenia patients show gray matter reduction is located in the left anterior cingulate gyrus.

**Figure 2:**
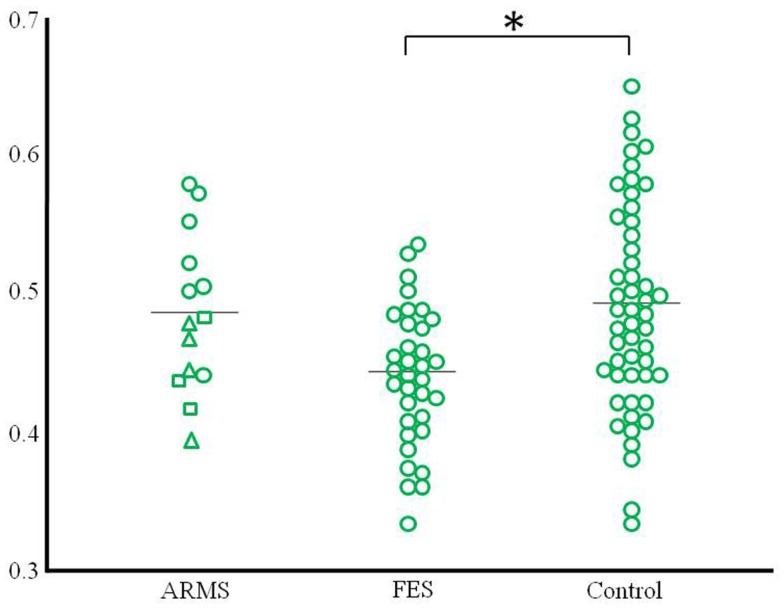
**Scatter plots of gray matter volume of the peak coordinate at the left anterior cingulate gyrus that revealed the difference between schizophrenia patients and controls**. The ARMS subjects were classified into three groups according to clinical outcome (square: ARMS who developed psychosis, triangle: ARMS with unknown outcome, circle: ARMS without transition to psychosis). *p < 0.05.

Compared with the healthy controls, the schizophrenia patients showed significant gray matter volume reduction in the left anterior cingulate gyrus (FWE-corrected *p* = 0.047) (Figures 1 and 2; Table 2). There was no difference between the ARMS subjects and the schizophrenia patients or the healthy controls.

**Table 2:** **Talairach coordinates for regions of reduced gray matter volume in the schizophrenia patients compared to the healthy controls**.

Region	Voxel	Peak coordinate			*T*	*p*
		*x*	*y*	*z*	
lt. anterior cingulate gyrus	631	−11	42	8	3.82	0.047

## Discussion

In this study, we performed VBM analyses using the DARTEL method to investigate gray matter change in early psychosis. In comparison to the healthy controls, first-episode schizophrenia patients showed significant gray matter reduction in the left anterior cingulate gyrus, but the ARMS subjects showed no significant difference in gray matter volume. This negative finding may be partly related to the heterogeneity of the ARMS subjects, as those with later transition to psychosis had a similar distribution of the cingulate gyrus gray matter volume to that in first-episode schizophrenia patients (Figure 2). These preliminary results are partly consistent with previous findings by Fornito et al. (2008), who reported that baseline differences of anterior cingulate gyrus distinguish between high-risk individuals who do and do not subsequently develop overt psychosis.

Neuroimaging studies comparing schizophrenia patients to healthy controls have shown evidence of morphological change in the anterior cingulate gyrus (Ellison-Wright et al., 2008; Shepherd et al., 2012). Gray matter volume reduction (Salgado-Pineda et al., 2003; Koo et al., 2008; Meisenzahl et al., 2008; Leung et al., 2011) and reduced cortical thickness (Schultz et al., 2010) in the anterior cingulate gyrus have been revealed by MRI studies in first-episode and neuroleptic-naïve patients to minimize the influence of neuroleptic medication or chronicity of the illness. In this study, gray matter volume reduction in the left anterior cingulate gyrus in the schizophrenia patients had no relationship with any effects of medication (data not shown).

One major aim of high-risk studies for psychosis has been to identify clinical and neurobiological predictors of future transition to psychosis, which would allow specific and targeted preventive strategies (McGorry et al., 2006); indeed, previous neuroimaging studies have identified such predictive markers. The VBM study by Pantelis et al. (2003) revealed the association between later transition and gray matter reduction in temporal and frontal regions predominantly in the right hemisphere and cingulate gyrus bilaterally in clinical high-risk subjects, which was largely replicated in an independent high-risk cohort (Borgwardt et al., 2007). Recent multi-center (Mechelli et al., 2011) and meta-analytic (Smieskova et al., 2010; Fusar-Poli et al., 2011) MRI studies on large numbers of high-risk subjects generally supported the assertion that brain morphological changes in the fronto-temporo-limbic regions, including the cingulate gyrus, already exist prior to the onset of psychosis. Although our data are clearly limited by the small sample size as discussed below, the distribution of the anterior cingulate gray matter volume (Figure 2) implies that ARMS subjects with later transition may have morphological changes of the cingulate gyrus to the same degree as those with overt schizophrenia. There has been debate about the risk-benefit ratio of antipsychotic treatment in prodromal patients (Woods et al., 2007; Weiser, 2011). However, given the hypothesized active brain pathology in the early phases of psychosis, which could affect the subsequent course of the illness (Birchwood et al., 1998), and the potential ameliorating effects of atypical antipsychotics for brain structural abnormalities (Lieberman et al., 2005; Girgis et al., 2006), intervention before the expression of frank psychosis may reduce neurobiological deterioration as well as the transition rate to psychosis (McGorry et al., 2002; McGlashan et al., 2006), especially in subjects with neurobiological risk markers.

The sample size of the current ARMS group (especially those who later developed psychosis) was small and some individuals dropped out during clinical follow-up (*N* = 4, unknown outcome group). Significant group differences in age (ARMS < schizophrenia and controls) might also have biased our results, although we used age as a controlling factor in all imaging analyses. In contrast to our prediction, we did not find significant brain morphological changes in the ARMS subjects, potentially due to the small sample size. It was also not possible to examine the relationship between brain morphology and clinical outcome (later transition) in our ARMS subjects statistically. In addition, direct comparison between the three groups using the ANOVA model with age and ICV as covariates failed to replicate significant group difference in the cingulate gyrus gray matter volume. Thus, further study with a larger well-defined sample is required to replicate and expand the current preliminary results.

In summary, the present study demonstrated significant gray matter reduction of the anterior cingulate gyrus in first-episode schizophrenia. We also suggested the possibility that such morphological change may exist prior to the onset of psychosis in some individuals, implying the potential role of neuroimaging methods in the prediction of future transition and effective intervention for high-risk subjects.

## Conflict of Interest Statement

The authors declare that the research was conducted in the absence of any commercial or financial relationships that could be construed as a potential conflict of interest.
